# Broad-spectrum antiviral activity of the synthetic rocaglate zotatifin against Mayaro virus and other viruses

**DOI:** 10.3389/fcimb.2026.1752166

**Published:** 2026-03-19

**Authors:** Patricia Valdés-Torres, Dalkiria Campos, Paola Elaine Galán-Jurado, Dalel Zegarra, Isaac Tuñon-Lorenzo, Félix González-Castillo, María Blanquer, Juan Castillo Mewa, Carmen Rivas, José González-Santamaría

**Affiliations:** 1 Grupo de Biología Celular y Molecular de Arbovirus, Departamento de Investigación en Virología y Biotecnología, Instituto Conmemorativo Gorgas de Estudios de la Salud, Panamá, Panama; 2 Programa de Maestría en Microbiología Ambiental, Universidad de Panamá, Panamá, Panama; 3 Programa de Desarrollo de las Ciencias Básicas (PEDECIBA), Universidad de la República, Montevideo, Uruguay; 4 Centro de Investigación en Medicina Molecular (CIMUS), Universidade de Santiago de Compostela, e Instituto de Investigación Sanitaria de Santiago de Compostela (IDIS), Santiago de Compostela, Spain; 5 Departamento de Investigación en Genómica y Proteómica, Instituto Conmemorativo Gorgas de Estudios de la Salud, Panamá, Panama; 6 Departamento de Biología Molecular y Celular, Centro Nacional de Biotecnología (CNB), CSIC, Madrid, Spain

**Keywords:** antiviral activity, arbovirus, rocaglates, virus, zotatifin

## Abstract

Viruses pose a significant threat to global public health, yet therapeutic options remain limited. Zotatifin, a synthetic rocaglate targeting eukaryotic initiation factor 4A (eIF4A), inhibits viral protein synthesis and potentially triggers interferon responses; however, its broad-spectrum antiviral potential remains unclear. We evaluated zotatifin’s cytotoxicity using MTT assays and assessed its antiviral activity through plaque-forming assays, Western blot analysis, immunofluorescence, and flow cytometry across multiple cell lines and virus strains. Zotatifin demonstrated superior tolerability compared to rocaglamide A and CR-1-31-B. The compound exhibited potent, dose-dependent inhibition of Mayaro virus, achieving a >4-log_10_ reduction in viral titers at 50 nM across all cell lines or virus strains tested. Zotatifin also effectively inhibited multiple arboviruses (Chikungunya, Una, and Zika viruses), influenza A virus, vesicular stomatitis virus, and vaccinia virus. Mechanistically, Zotatifin down-regulated viral protein synthesis for all viruses tested, and RT-qPCR analysis revealed activation of the type I interferon pathway in treated cells. Collectively, these results demonstrate that zotatifin exhibits broad-spectrum antiviral activity against five virus families, through different molecular mechanisms, supporting its potential therapeutic use as a pan-antiviral drug in humans.

## 1 Introduction

Despite the considerable risks that viruses pose to human health, effective antiviral drugs and licensed vaccines remain unavailable for many viral pathogens. This therapeutic gap has prompted researchers to explore novel antiviral strategies, particularly those targeting host factors essential for viral replication. Host-directed antivirals (HDA) function by blocking cellular proteins or pathways required during any phase of the viral replication cycle. Since many viruses exploit shared cellular pathways and factors, a single HDA can exhibit activity against viruses from distinct families. This approach offers several key advantages, including broad-spectrum antiviral activity and reduced susceptibility to viral resistance. Rocaglates, a class of naturally occurring compounds derived from *Aglaia* plants, have emerged as promising broad-spectrum antiviral agents with significant therapeutical potential (Agarwal et al., 2021; Harneti and Supratman, 2021).

Rocaglates exert antiviral properties primarily through interaction with eukaryotic initiation factor 4A (eIF4A), a DEAD-box RNA helicase and key component of the protein synthesis machinery (Greger, 2022; Li et al., 2023; Li-Weber, 2015). eIF4A is essential for the translation of structured mRNAs, including many viral transcripts that contain complex 5’ untranslated regions (UTRs) (Taroncher-Oldenburg et al., 2021). By targeting eIF4A, rocaglates effectively block viral protein synthesis, thereby inhibiting viral replication while showing selectivity for viral over cellular mRNAs (Taroncher-Oldenburg et al., 2021).

The natural rocaglate silvestrol has demonstrated remarkable antiviral activity against multiple viral pathogens, including Middle East respiratory syndrome coronavirus (MERS-CoV), human coronavirus 229E (HCoV-229E), influenza A virus (IAV), Ebola virus (EBOV), hepatitis E virus (HEV), and Zika virus (ZIKV) (Biedenkopf et al., 2017; Elgner et al., 2018; Glitscher et al., 2018; Muller et al., 2018; Slaine et al., 2017; Todt et al., 2018). Building on these findings, researchers have developed synthetic rocaglates with comparable efficacy. For instance, the synthetic compound CR-1-31-B has been reported to exhibit antiviral activity like silvestrol (Müller et al., 2020).

More recently, zotatifin (eFT226), another synthetic rocaglate (Ernst et al., 2020), has shown particularly promising results. This compound effectively controls replication of various coronaviruses and reduces SARS-CoV-2 viral infectivity (Gordon et al., 2020; Obermann et al., 2022). Importantly, zotatifin exhibits reduced cytotoxicity toward immune cells compared to other rocaglates and has entered clinical trials for cancer (ClinicalTrials.gov: NCT04092673) and COVID-19 (ClinicalTrials.gov: NCT04632381) (Obermann et al., 2022). Recent studies have revealed that zotatifin may have other mechanisms of action beyond eIF4A inhibition such as the induction of the interferon (IFN) pathway, which may contribute to its antiviral efficacy (Zhao et al., 2023). However, despite these encouraging findings, zotatifin’s antiviral spectrum against viral families beyond coronaviruses remains largely unexplored.

In this study we have evaluated zotatifin’s potential to control viral replication across diverse viral families, including representatives from the *Togaviridae* (Mayaro, Chikungunya, and Una viruses), *Flaviviridae* (Zika virus), *Orthomyxoviridae* (influenza A virus), *Rhabdoviridae* (vesicular stomatitis virus), and *Poxviridae* (vaccinia virus). Our results demonstrated that zotatifin exhibits broad-spectrum antiviral activity against five virus families, supporting its potential therapeutic use as a pan-antiviral drug in humans.

## 2 Materials and methods

### 2.1 Cell lines, virus strains, and reagents

Human dermal fibroblasts (HDFs, Cat. # PCS-201-012), human immortalized microglial cells (HMC3, Cat. # CRL-3304), human non-small cell lung cancer A549 cells (CCL-185), BSC40 (Cat. # CRL-2761), and Vero-E6 cells (CRL-1586) were obtained from the American Type Culture Collection (ATCC, Manassas, VA, USA). HDFs and A549 cells were cultivated in Dulbecco’s Modified Eagle Medium (DMEM), HMC3 cells in Eagle’s Minimum Essential Medium (EMEM), and Vero-E6 in Minimal Essential Medium (MEM). All media were supplemented with 10% fetal bovine serum (FBS), 2 mM L-glutamine, and 1% penicillin-streptomycin solution. Cells were maintained at 37 °C in a humidified atmosphere with 5% CO2.

The Mayaro virus (MAYV) strains AVR0565 (Peru), TRVL 4675 (Trinidad and Tobago), BeH256 (Brazil), and D218 (Suriname), as well as Una virus (UNAV) BT 1495-3 (Panama) were obtained from the World Reference Center for Emerging Viruses and Arboviruses (WRCEVA) at the University of Texas Medical Branch (UTMB) in Galveston, Texas, USA and were kindly provided by Dr. Scott Weaver. Chikungunya (CHIKV, Panama_256137) and Zika (ZIKV, 259249) viruses were isolated from patients in Panama. The recombinant GFP-expressing vesicular stomatitis virus (rVSV-GFP) and influenza A virus (PR8-GFP) were provided by Dr. Adolfo García-Sastre (Icahn School of Medicine at Mount Sinai in New York, USA). Vaccinia virus (VV) WR strain was provided by Dr. Mariano Esteban (Centro Nacional de Biotecnología, CSIC, Spain). All virus strains were propagated in Vero-E6 or A549 cells, titrated, aliquoted, and stored at -80 °C as previously reported (Valdes-Torres et al., 2022). The rocaglates rocaglamide A (Cat. # HY-19356), CR1-31-B (Cat. # HY-136453), and zotatifin (also known as eFT226, Cat. # HY-112163) were purchased from MedChemExpress (New Jersey, USA). All compounds were dissolved in DMSO and stored at -20°C until use. Working solutions were prepared in appropriate culture media at indicated concentrations immediately before use.

### 2.2 Cell viability assay

Cytotoxicity of rocaglates was evaluated using the MTT assay as previously described (Valdes-Torres et al., 2022). Briefly, cells were seeded in 96-well plates at density of 1x10^4^ cells per well and allowed to adhere overnight. Cells were then treated with different concentrations of rocaglamide A, CR1-31-B, or zotatifin. After 24 hours of incubation, cells were treated with 20 μL of 5 mg/ml solution of 3-(4,5-dimethyl-2-thiazolyl)-2,5-diphenyl tetrazolium bromide (MTT, Sigma-Aldrich, St. Louis, Missouri, USA) and incubated for an additional 2 h. The formazan crystals were then dissolved in 100 μL DMSO, and absorbance at 570 nm was measured using a microplate reader (BioTek, Winooski, VT, USA). The results are presented as the percentage of viable cells relative to DMSO-treated control cells.

### 2.3 Plaque-forming assay

Vaccinia virus was harvested from infected cells by three cycles of freezing/thawing, sonication and clarification by centrifugation. Clarified supernatants and virus titers in cell culture supernatants were quantified using plaque-forming assays as previously reported (Valdes-Torres et al., 2022). Briefly, 10-fold serial dilutions of infected samples were used to infect confluent Vero-E6, A549 or BSC40 cells in 12-well plates. After 1 hour of incubation at 37 °C, supernatants were removed, and the cells were overlaid with a solution of 1% agar in MEM supplemented with 2% FBS. Plates were incubated at 37 °C until plaques were visible (24–72 h depending on the virus). The agar overlay was removed, and the cells were fixed with 4% formaldehyde in PBS for 20 minutes, then stained with 2% crystal violet in 30% methanol. Plaques were counted manually, and the virus titers were expressed as plaque-forming units per milliliter (PFU/ml). The limit of detection (LOD) for all viruses was 10 PFU/ml. Only wells containing 10–100 plaques were used for titer calculations to ensure accuracy.

### 2.4 Virus infection and cytopathic effect assays

HDFs, HMC3, and A549 cells were seeded in 12- or 24-well plates at appropriate densities and allowed to adhere overnight. Cells were pretreated with DMSO (vehicle control), rocaglamide A, CR-1-31-B, or zotatifin at indicated concentrations for 2 h at 37 °C. Following pretreatment, cells were infected with MAYV, CHIKV, UNAV, ZIKV, PR8-GFP, or rVSV-GFP at specified multiplicities of infection (MOI). After virus adsorption, the inoculum was removed and fresh medium containing the respective compounds was added. At the indicated hours post infection, cell culture supernatants were collected for virus titration, and cells were processed for protein analysis. When the experiments were carried out with VV, cells were collected to be used or either virus titration for protein analysis, as indicated. For cytopathic effect assays, cells were pre-treated with either DMSO or zotatifin (50 nM) for 2 h. Subsequently, cells were infected with MAYV (AVR0565 strain) and maintained in the presence or absence of zotatifin for 48 h post-infection. Cells were fixed with formaldehyde and stained with crystal violet using the protocol described above. Cell morphology was examined using an inverted microscope equipped with an MCI70-HD camera (Leica, Buffalo Grove, IL, USA).

For time-of-addition studies, the following protocols were used:

- Pretreatment assay: cells were treated with zotatifin for 2 h, then washed with PBS and infected with virus in compound-free medium.
- Binding assay: cells were cooled at 4 °C, incubated with virus and treated simultaneously with zotatifin for 1 h. Following incubation, cells were washed with PBS and incubated in compound-free medium.
- Entry assay: cells were infected at 4 °C for 1 h, transferred to 37 °C, and zotatifin was added for 2 h. Following incubation, cells were washed with PBS and incubated in compound-free medium.
- Post-entry assay: zotatifin was added at 2 h post-infection at 37 °C and maintained until harvest at 24 h.

In all assays, viral titers were quantified at 24 hours post-infection.

### 2.5 Flow cytometry assay

A549 cells were seeded in 6-well plates and treated with DMSO or zotatifin (50 nM) for 2 h, then infected with PR8-GFP or rVSV-GFP at MOI 0.5 or 5.0 PFU/cell and then incubated in the presence or absence of zotatifin. Twenty-four hours later, the cells were harvested by tripsinization, washed with PBS, and fixed with 2% paraformaldehyde for 20 minutes at room temperature. Fixed cells were washed twice and resuspended in 250 μL PBS. Samples were analyzed using a CytoFLEX S flow cytometer (Beckman Coulter, Florida, USA) by acquiring at least 10,000 events per sample. GFP fluorescence was detected using 488-nm excitation and 525–540 nm emission filters. Data were analyzed using CytoExpert software (v2.4.0.28) with appropriate compensation controls.

### 2.6 Western blot assay

Protein extracts were obtained from mock- or virus-infected cells treated or untreated with zotatifin, using Laemmli buffer containing 10% dithiothreitol (Bio-Rad, Hercules, CA, USA). The proteins were then separated using SDS-PAGE on 10-12% Bis-Tris gels, and transferred to nitrocellulose membranes. Membranes were blocked with 5% nonfat milk in T-TBS buffer for 30 minutes at room temperature, then incubated overnight at 4 °C with primary antibodies: rabbit polyclonal anti-E1 (1:1000) and rabbit polyclonal anti-nsP1 (1:1000) for MAYV, UNAV, and CHIKV (Llamas-González et al., 2019); anti-nsP2 of CHIVK (Cat. # GTX135188, GeneTex, USA); anti-NP of IAV (1:1000) (GTX125989, USA); anti-G of VSV (kindly provided by Dr. Iván Ventoso (Centro de Biología Molecular Severo Ochoa, Spain); and anti-GAPDH (1:1000, Cat. # VMA00046, Bio-Rad); or anti-β-actin (1:1000, Cat. # VMA00048, Bio-Rad). After washing three times with T-TBS, membranes were incubated with HRP-conjugated goat anti-rabbit (Cat. # 926-80011, LI-COR, Lincoln, NE, USA) or goat anti-mouse (Cat. # 926-80010, LI-COR, Lincoln, NE, USA) secondary antibodies for 1 h at room temperature. Chemiluminescent signals were detected using SignalFireTM ECL Reagent (Cell Signaling Technology, Danvers, MA, USA) and visualized using a C-Digit scanner (LICOR, Lincoln, NE, USA) or a film system.

### 2.7 Immunofluorescence assay

A549 cells grown on glass coverslips in 24-well plates were treated with or without zotatifin (50 nM) for 2 h, then infected with the MAYV ARV0565 strain at a MOI of 1 in presence or absence of the compound. At 24 h post infection, cells were fixed with 2% paraformaldehyde for 20 minutes, permeabilized with Triton X-100 for 10 minutes, and blocked with 5% bovine serum albumin as previously described (Sugasti-Salazar et al., 2021). Cells were stained with rabbit anti-E1 or anti-nsP1 primary antibodies (1:100) overnight at 4 °C, followed by incubation with Alexa Fluor 568 goat anti-rabbit secondary antibody (1:1000, Invitrogen, Carlsbad, USA) for 1 h at room temperature. Coverslips were mounted using Prolong Diamond Antifade with DAPI (Invitrogen, Carlsbad, USA). Images were acquired using an FV3000 Fluoview confocal microscope (Olympus, USA).

### 2.8 Gene expression analysis by RT-qPCR

Total RNA was extracted from A549 cells treated with or without zotatifin (50 nM) for 8 h using the RNeasy kit (QIAGEN, Valencia, CA, USA), according to the manufacturer´s instructions. Single-stranded cDNA was synthesized from 1 μg of RNA using a High-Capacity cDNA Reverse Transcription kit. RT-qPCR was performed using Power SYBR Green PCR Master Mix in a QuantiStudio 5 thermocycler (Applied Biosystems, Foster City, CA, USA) and the primers indicated in **Table 1**, as previously described (Valdes-Torres et al., 2022). Relative mRNA expression was calculated using the β-actin gene for normalization with the ΔΔCT method (Livak and Schmittgen, 2001).

**Table 1:** Primers used in this study.

Gene	Primer sequences (5’-3’)
*IFNα*	Forward: GCCTCGCCCTTTGCTTTACT
	Reverse: CTGTGGGTCTCAGGGAGATCA
*DDX58*	Forward: TGTTCTCAGATCCCTTGGATG
	Reverse: CACTGCTCACCAGATTGCAT
*ISG15*	Forward: GAGAGGCAGCGAACTCATCT
	Reverse: CTTCAGCTCTGACACCGACA
*MxA*	Forward: GGTGGTGGTCCCCAGTAATG
	Reverse: ACCACGTCCACAACCTTGTCT
*TLR3*	Forward: TGGGACCAAGGCAAAGGAGT
	Reverse: TTCTCTTGGTTGGGCCACCT
*β-actin*	Forward: AGAGCTACGAGCTGCCTGAC
	Reverse: AGCACTGTGTTGGCGTACAG

### 2.9 Data analysis

All experiments were performed at least twice in triplicate. Data are presented as mean ± standard deviation. Statistical comparisons were performed using one-way ANOVA followed by Dunnettt’s *post hoc* test for multiple comparisons against control, or unpaired t-test for pairwise comparisons. A *p*-value < 0.05 was considered statistically significant. All statistical analysis and graphics were generated using GraphPad Prism software (version 10.6.0 for Mac; GraphPad Software, San Diego, CA, USA).

## 3 Results

### 3.1 Zotatifin potently controls Mayaro virus replication

**Figure 1:**
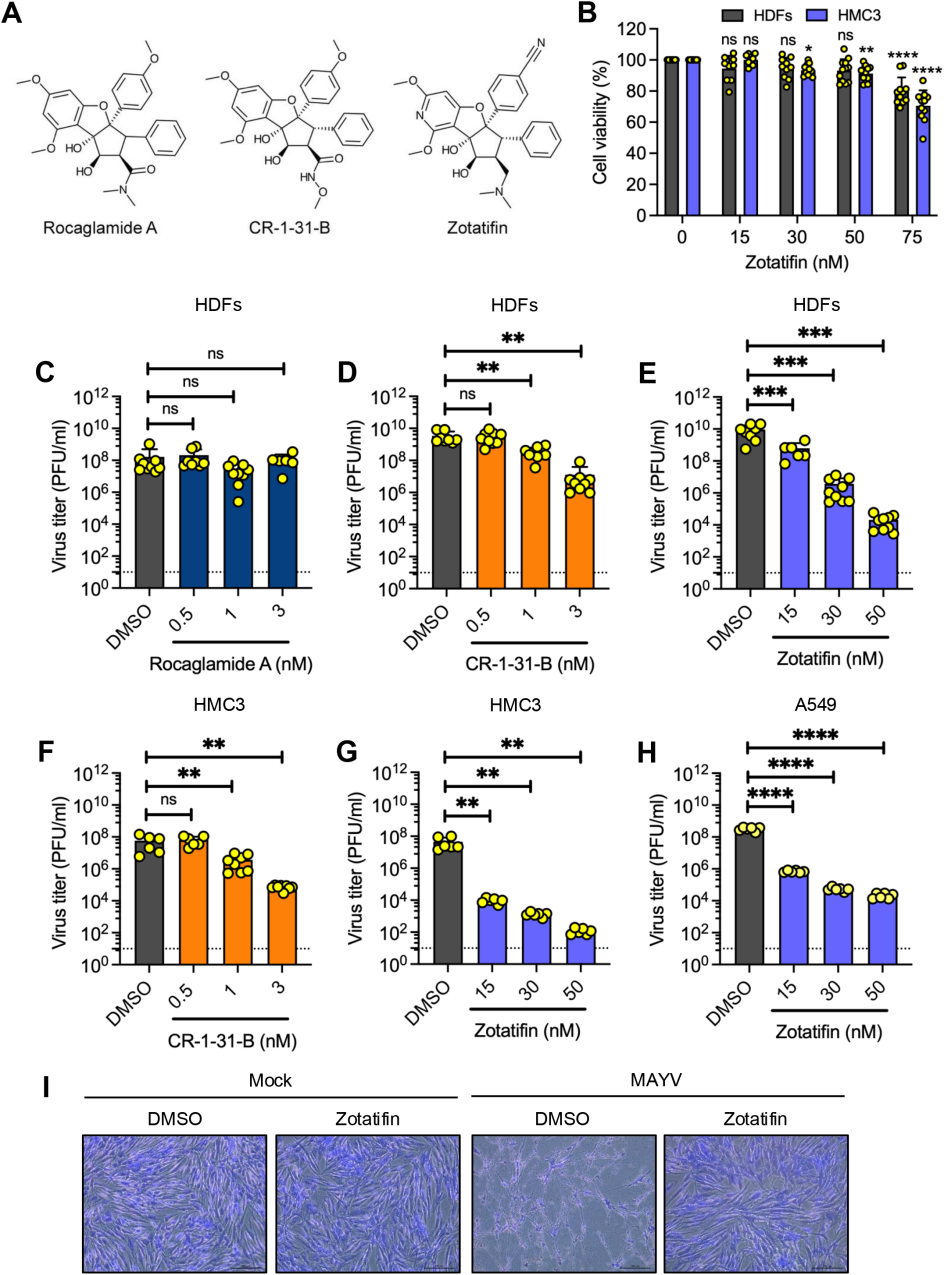
Zotatifin demonstrates dose-dependent inhibition of Mayaro virus (MAYV) replication across multiple cell lines. **(A)** Chemical structures of rocaglate compounds evaluated in this study. **(B)** Cell viability assessment in HDFs and HMC3 cells following 24 h treatment with increasing zotatifin concentrations, determined using the MTT assay. **(C–H)** Antiviral efficacy evaluation across three cell lines. HDFs **(C–E)**, HMC3 **(F–G)**, and A549 cells **(H)** were pretreated with rocaglates for 2 h, infected with MAYV strain AVR0565 (MOI = 1). Following 24 h incubation with compounds, viral progeny was quantified by plaque-forming assay. The dashed horizontal line indicates the limit of detection (10 PFU/ml). Data shown for rocaglamide (blue bars), CR-1-31-B (orange bars), and zotatifin (violet bars). **(I)** Morphological analysis of MAYV-infected HDFs with zotatifin treatment (50 nM). Cells were fixed at 48 h post-infection with a 4% formaldehyde solution and stained with a 2% crystal violet solution. Scale bar: 100 μm. Values represent mean ± standard deviation from three independent experiments, each performed in triplicate **(C–H)** o quadruplicate **(B)**. Statistical analysis was performed using a one-way ANOVA, followed by a Dunnett’s *post hoc* test comparing treated groups to vehicle control. Significance levels: ns, not significant; *p < 0.05; **p < 0.01; ***p < 0.001; and ****p < 0.0001.

To evaluate the cytotoxicity profile of zotatifin relative to other rocaglates, we compared it with the natural compound rocaglamide A and the synthetic analog CR-1-31-B (**Figure 1A**). We selected two clinically relevant cell models: primary human dermal fibroblasts (HDFs), which represent a physiological relevant dermal target for arbovirus infection (Hoover and Fredericksen, 2014; Sugasti-Salazar et al., 2021), and human immortalized microglia cells (HMC3), which model for neurological pathogenesis. Both cell lines are highly permissive for Mayaro virus (MAYV) infection (Campos et al., 2024; Sugasti-Salazar et al., 2021), an emerging alphavirus in the *Togaviridae* family that poses a potential threat to the Americas (Wei et al., 2024). Cytotoxicity was assessed using the MTT assay.

Both rocaglamide A and CR-1-31-B exhibited notable toxicity at doses exceeding 5 nM in both cell lines (**Supplementary Figures 1A, B**; <80% cell viability). In contrast, zotatifin demonstrated favorable tolerability, maintaining approximately 80% cell viability even at 50 nM (**Figure 1B**), a 10-fold higher concentration than the toxic threshold observed for the other rocaglates (**Supplementary Figures 1A, B**).

We next evaluated the antiviral potential of zotatifin against MAYV and compared its efficacy with other rocaglates. HDFs and HMC3 cells were pretreated with the indicated concentrations of rocaglates for 2 h before being infected with MAYV at a MOI of 1. Viral titers in culture supernatants were evaluated 24 h post-infection using plaque-forming assays. Rocaglamide A treatment did not significantly reduce MAYV replication at non-toxic concentrations (**Figure 1C**). In contrast, CR-1-31-B inhibited MAYV replication by 2–3 log_10_ in both HDFs and HMC3 cells (**Figures 1D, F**). Most notably, zotatifin demonstrated potent inhibition of MAYV replication in both cell lines, achieving greater than 4-log_10_ reduction in viral titers at 50 nM (**Figures 1E, G**).

To validate these findings across different cell types, we evaluated zotatifin’s antiviral efficacy in human A549 lung epithelial cells, a well-established cell line for antiviral screening (Lai et al., 2020). Treatment with zotatifin resulted in a significant dose-dependent reduction in viral titers (**Figure 1H**), without detectable cytotoxicity (**Supplementary Figure 1C**). Importantly, zotatifin retained its antiviral activity when administered 1 h post-viral absorption, confirming therapeutic efficacy even after infection initiation (**Supplementary Figure 2**).

As with other alphaviruses, MAYV induces robust cytopathic effects in several cell lines, including HDFs (Sugasti-Salazar et al., 2021). Microscopic examination at 48 h post-infection revealed that zotatifin treatment completely protected HDFs against MAYV-induced cytopathic effects (**Figure 1I**), maintaining cell morphology like that of uninfected controls. Overall, these data demonstrate that zotatifin effectively controls MAYV replication in HDFs, HMC3, and A549 cells.

### 3.2 Zotatifin reduces the expression of MAYV E1 and nsP1 proteins

**Figure 2:**
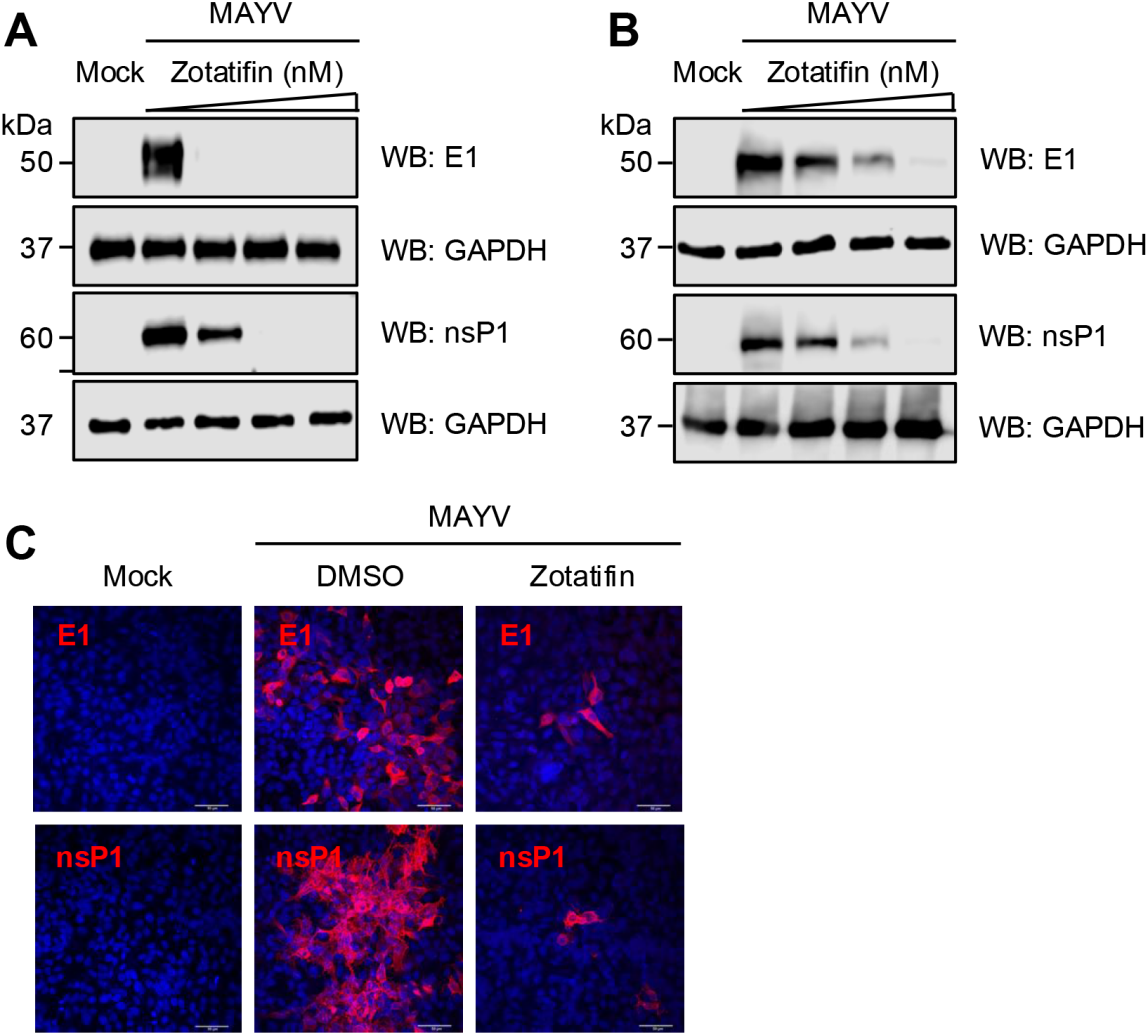
Zotatifin reduces MAYV E1 and nsP1 protein levels in a dose-dependent manner. HDFs **(A)** or A549 cells **(B)** were pretreated with zotatifin at concentrations of 15, 30, and 50 nM for 2 h. Cells were subsequently infected with MAYV strain AVR0565 (MOI = 1) and maintained in the presence of zotatifin throughout the infection period. At 24 h post-infection, viral protein levels were assessed by Western blot analysis using specific antibodies against MAYV E1 and nsP1 proteins. Glyceraldehyde-3-phosphate dehydrogenase (GAPDH) served as the loading control. Representative blots from three independent experiments are shown. **(C)** A549 cells were treated with 50 nM zotatifin or vehicle control and infected with MAYV strain AVR0565. At 24 h post-infection, cells were fixed and processed for immunofluorescence microscopy using antibodies specific for MAYV E1 and nsP1 proteins. Representative immunofluorescence images from at least 10 fields across two independent experiments are presented. Scale bar: 50 μm.

Zotatifin is a potent inhibitor of the RNA helicase eIF4A, which plays a critical role in protein translation initiation (Ernst et al., 2020). Therefore, we analyzed the synthesis of MAYV E1 and nsP1 proteins following zotatifin treatment. Western blot analysis revealed that zotatifin treatment reduced the synthesis of both viral proteins in a dose-dependent manner in both HDFs (**Figure 2A**) and A549 cells (**Figure 2B**). Similar results were detected using immunofluorescence assays. We observed a reduction in the levels of MAYV E1 and nsP1 proteins in the A549 cells treated with 50 nM zotatifin (**Figure 2C**), a concentration that was well tolerated by the cells (**Supplementary Figure 1C**). These results indicated that zotatifin significantly downregulates MAYV E1 and nsP1 protein expression.

### 3.3 Zotatifin exhibits strain-independent antiviral activity against MAYV

**Figure 3:**
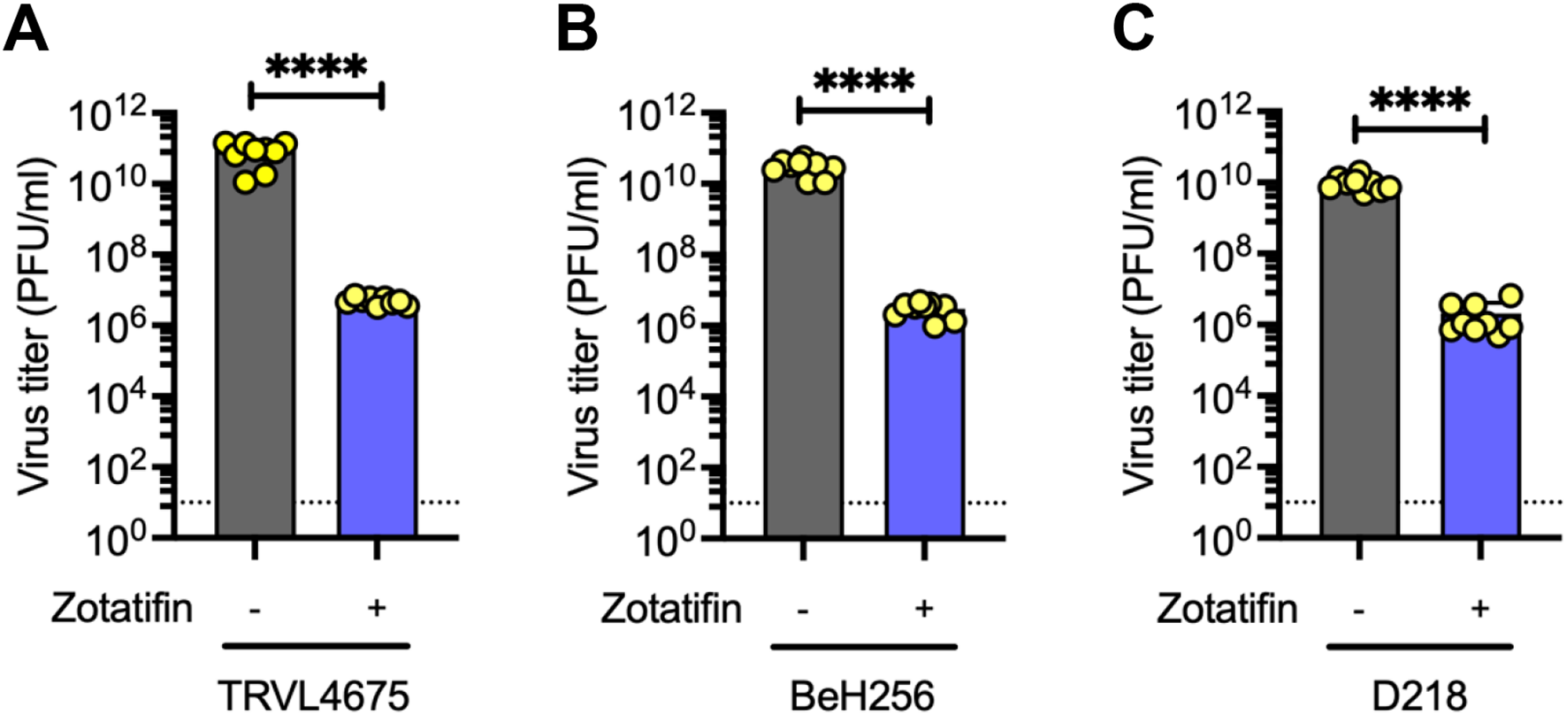
Zotatifin inhibits MAYV replication in a strain-independent manner. HDFs were pretreated with zotatifin (50 nM) and then infected with the following MAYV strains: TRVL4675 (Trinidad and Tobago) **(A)**, BeH256 (Brazil) **(B)**, or D218 (Surinam) **(C)**. After 24 h of infection in presence or absence of the compound, viral progeny production was assessed using a plaque-forming assay. The dashed horizontal line indicates the limit of detection (10 PFU/ml). Data represent mean ± standard deviation from three independent experiments performed in triplicate. Statistical significance was determined by unpaired Student t-test. Significance level: ****p < 0.0001.

We then decided to study zotatifin’s ability to inhibit the replication of different MAYV strains isolated from various regions in Latin America. We treated HDFs with or without 50 nM zotatifin for 2 h and then infected with MAYV strains from Trinidad and Tobago (TRVL 4675), Brazil (BeH256), or Suriname (D218). Zotatifin significantly reduced viral titers by approximately 4–5 log_10_ for all tested MAYV strains (**Figures 3A–C**), indicating strain-independent antiviral efficacy.

### 3.4 Zotatifin acts through different mechanisms in the MAYV life cycle

**Figure 4:**
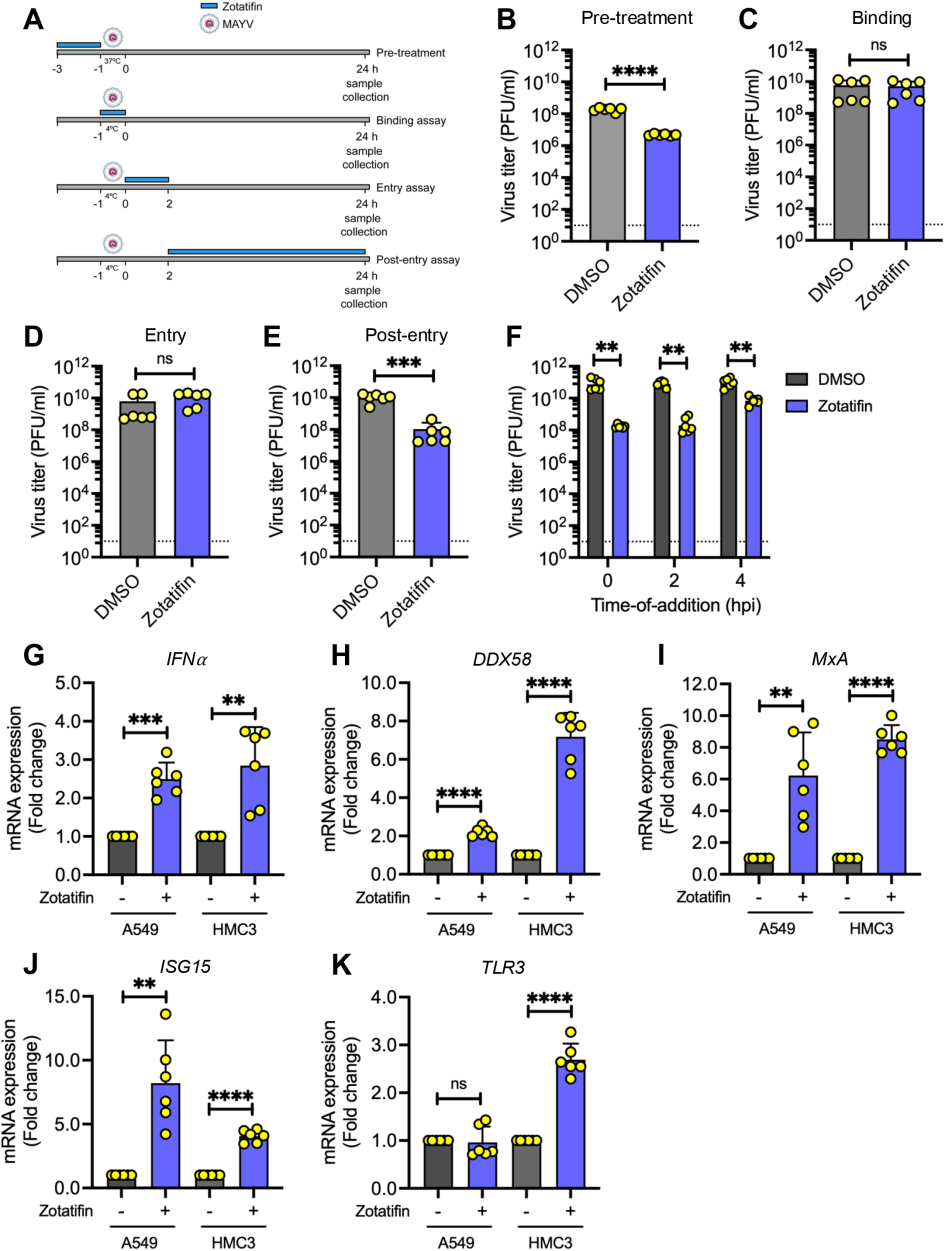
Zotatifin inhibits MAYV replication through stage-specific mechanisms and enhances innate immune responses. **(A)** Schematic representation of the experimental design for viral cycle analysis, showing the pre-treatment, binding, entry, and post-entry assays protocols. HDFs were treated with zotatifin (50 nM) and infected with MAYV (AVR0565 strain). Viral titers in culture supernatants were quantified by plaque-forming assay for: **(B)** pre-treatment assay (cells pre-treated with zotatifin before infection), **(C)** binding assay (zotatifin present during viral adsorption), **(D)** entry assay (zotatifin added during viral entry phase), and **(E)** post-entry assay (zotatifin added after viral internalization). **(F)** Time-of-addition analysis. HDFs were infected with MAYV and zotatifin (50 nM) was added at indicated time points post-infection. Viral titers were determined by plaque-forming assay at 24 h post-infection. The dashed horizontal line indicates the limit of detection (10 PFU/ml). **(G–K)** Zotatifin upregulates interferon and interferon-stimulated gene expression. A549 and HMC3 cells were treated or untreated with 50 nM zotatifin for 8 h. Relative mRNA expression levels were analyzed by RT-qPCR for: **(G)** interferon-α (*IFNα*), **(H)** RIG-I-like receptor (*DDX58*), **(I)** myxovirus resistance protein A (*MxA*), **(J)** interferon-stimulated gene 15 (*ISG15*), and **(K)** Toll-like receptor 3 (*TLR3*). Expression levels were normalized to β-actin using the ΔΔCt method. Data represent mean ± standard deviation from two independent experiments performed in triplicate. Statistical significance was determined by unpaired Student t-test. Significance levels: ns, not significant; **p < 0.01; ***p < 0.001; ****p < 0.0001.

Time-of-addition studies were conducted to determine zotatifin’s mechanism of action against MAYV (**Figure 4A**). Pretreatment of HDFs with zotatifin (50 nM) for only 2 h prior to infection reduced MAYV titers by approximately 2 log_10_ (**Figure 4B**, *p* < 0.01), suggesting the activation of cellular antiviral pathways by the compound. During the binding assay, in which zotatifin was only present during virus attachment at 4 °C, no significant reduction in viral titers was observed (**Figure 4C**), indicating that zotatifin does not interfere with virus binding. Similarly, the entry assay showed no significant effect when zotatifin was present only during the 2 h entry period (**Figure 4D**). However, adding zotatifin 2 h post-infection and maintaining it for 24 h resulted in a significant reduction in viral titer (**Figure 4E**, *p* < 0.001), which is consistent with the inhibition of viral protein synthesis through eIF4A targeting. These results suggest that zotatifin may act through different mechanisms: activation of cellular antiviral responses during pretreatment and direct inhibition of viral protein synthesis during the post-entry phase (**Supplementary Figure 3**).

To assess the therapeutic window for zotatifin intervention, we examined whether the compound retains activity after viral infection has been established. HDFs were infected with MAYV, and following 1 h of virus adsorption, zotatifin was applied at the indicates times post-infection. As shown in **Figures 4F**, zotatifin effectively inhibited MAYV when added up to 4 h post-infection, demonstrating that the compound maintains antiviral efficacy even after viral infection initiation.

The antiviral effect observed with short-term zotatifin pretreatment, combined with previous reports of zotatifin-induced interferon-stimulated genes (ISGs) (Zhao et al., 2023), prompted us to investigate whether zotatifin activates the type I interferon (IFN) pathway. We treated A549 and HMC3 cells with 50 nM zotatifin for 8 h and analyzed the expression of key IFN pathway genes by RT-qPCR. Zotatifin treatment resulted in significant upregulation of *IFNα*, *DDX58*, *MxA*, and *ISG15*, in both cell lines compared to DMSO-treated controls (**Figures 4G–K**). These results indicate that zotatifin can activate type I IFN signaling, providing a possible mechanistic basis for its antiviral efficacy when used as a pretreatment (**Supplementary Figure 3**).

### 3.5 Zotatifin demonstrates broad-spectrum activity against arboviruses

**Figure 5:**
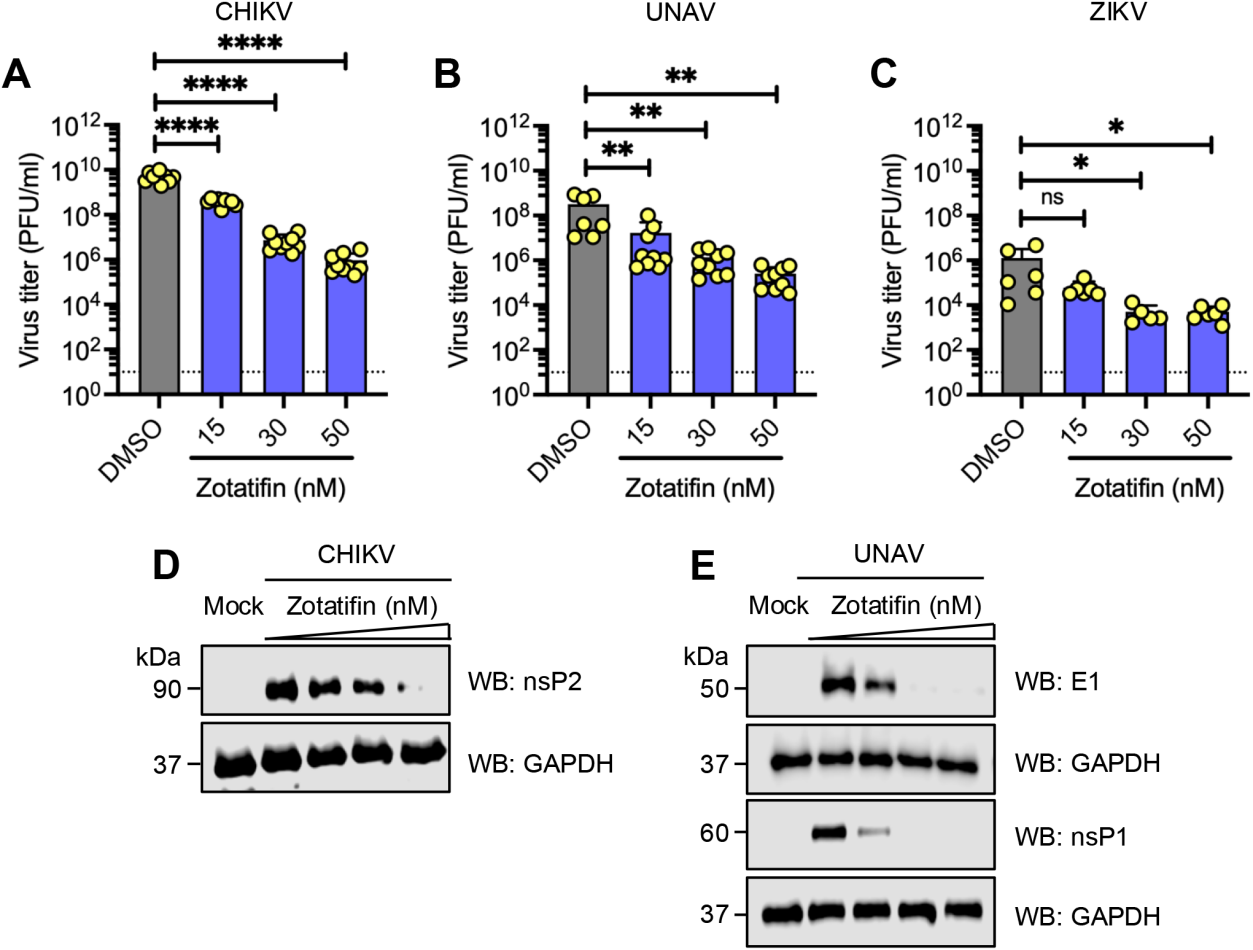
Zotatifin inhibits the replication of the arbovirus Chikungunya, Una, and Zika, in a dose-dependent manner. **(A–C)** Dose-dependent inhibition of viral progeny production. HDFs were pretreated with zotatifin at the indicated concentrations, followed by infection with **(A)** CHIKV, **(B)** UNAV, or **(C)** ZIKV, as previously described. At 24 h post-infection, viral progeny titers in cell culture supernatants were quantified by plaque-forming assay. The dashed horizontal line indicates the limit of detection (10 PFU/ml). Data represent mean ± standard deviation from three independent experiments performed in triplicate. Statistical analysis was carried out using a one-way ANOVA followed by a Dunnett’s *post hoc* test comparing treated samples to untreated controls. Significance levels: *p < 0.05; **p < 0.01; ****p < 0.0001. **(D, E)** Zotatifin reduces viral protein expression. HDFs were pretreated with zotatifin (15, 30, and 50 nM) and subsequently infected with CHIKV or UNAV. Viral protein levels of the nsP2 **(D)**, E1, and nsP1 **(E)** proteins were analyzed using Western blot assays. Glyceroaldehyde-3-phosphate dehydrogenase (GAPDH) served as a loading control. Representative images from three independent experiments are shown.

To evaluate the antiviral breadth of zotatifin, we tested its efficacy against several medically important arboviruses: Chikungunya (CHIKV), Una (UNAV), and Zika (ZIKV). HDFs were treated with zotatifin (50 nM) and then infected with each virus at a MOI of 1. Zotatifin significantly reduced viral titers in a dose-dependent manner for CHIKV (4-log_10_ reduction at 50 nM, *p* < 0.0001), UNAV (2.35 log_10_ at 50 nM, *p* < 0.01), and ZIKV (2.4 log_10_ reduction at 50 nM, *p* < 0.05) (**Figures 5A–C**).

Finally, we evaluated whether zotatifin treatment affected the synthesis of viral proteins. We observed a reduction in CHIKV nsP2 and UNAV E1, and nsP1 protein levels in response to zotatifin in a dose-dependent manner (**Figures 5D, E**). These results demonstrate that zotatifin exhibits broad-spectrum antiviral activity against multiple arbovirus members.

### 3.6 Zotatifin inhibits viruses from *Orthomyxoviridae*, *Rhabdoviridae*, and *Poxviridae* families

**Figure 6:**
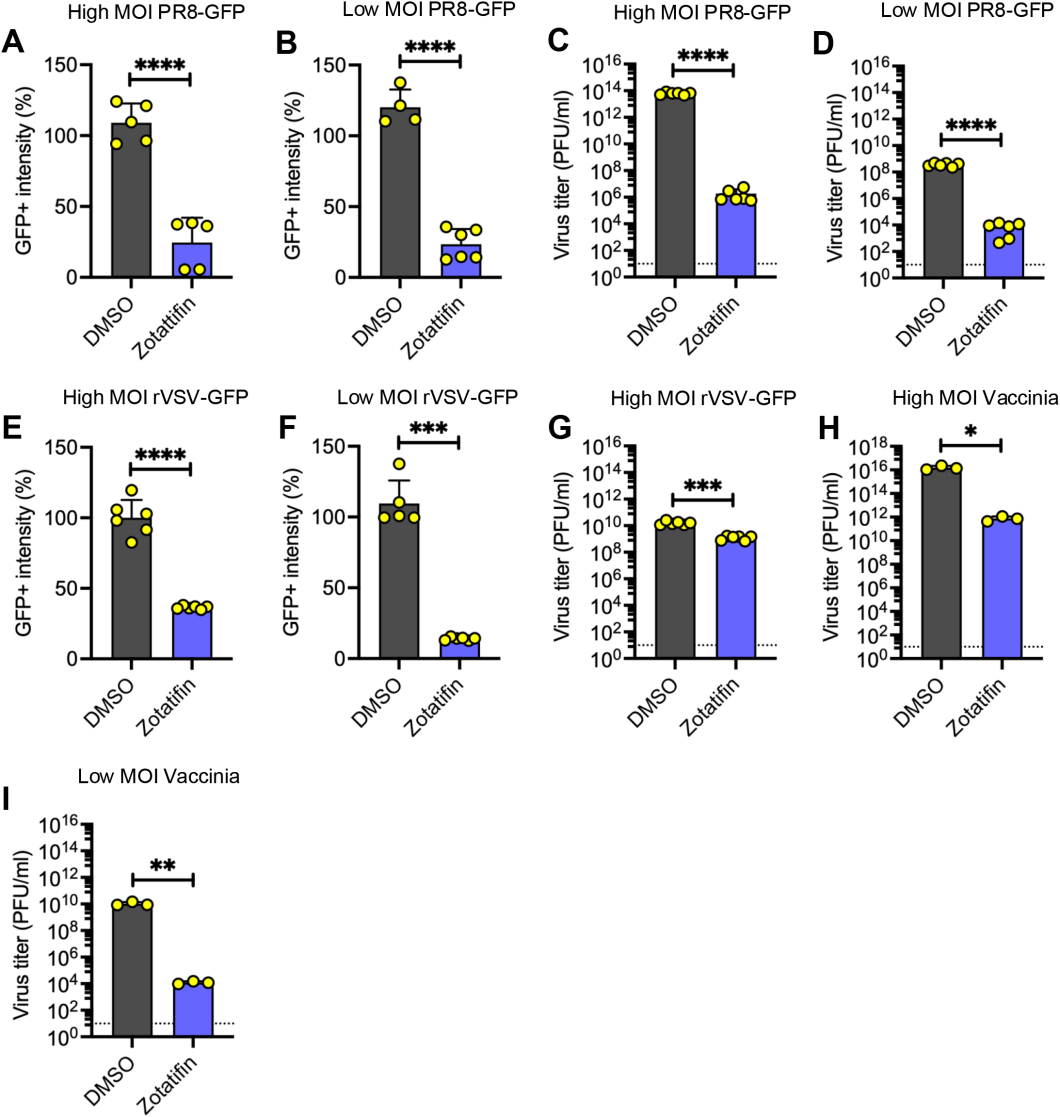
Zotatifin inhibits the replication of influenza A, vesicular stomatitis, and vaccinia viruses. A549 cells were pretreated with zotatifin, then infected with PR8-GFP **(A–D)**, rVSV-GFP **(E–G)** or vaccinia **(H, I)** viruses at low (0.5) or high (5) multiplicity of infection (MOI). At 24 h post-infection, the intensity of GFP, and the quantity of viral progeny in PR8-GFP and rVSV-GFP infected cells were assessed using flow cytometry, and a plaque-forming assay. Virus titers in cell lysates of vaccinia virus infected cells were determined by plaque assay. The dashed horizontal line indicates the limit of detection (10 PFU/ml). Data represent mean ± standard deviation from two independent experiments performed in triplicate. Statistical analysis was carried out using a one-way ANOVA followed by a Dunnett’s *post hoc* test or an unpaired Student t-test. Significance levels: ns, not significant; *p < 0.05; **p < 0.01; ***p < 0.001; and ****p < 0.0001.

To further evaluate zotatifin’s broad-spectrum potential, we assessed its efficacy against three phylogenetically distinct viral families: influenza A (IAV; *Orthomyxoviridae* family), vesicular stomatitis (VSV; *Rhabdoviridae* family), and vaccinia (VV; *Poxviridae* family). A549 cells were pretreated with zotatifin or vehicle control, followed by infection with recombinant reporter viruses expressing GFP (PR8-GFP for IAV and rVSV-GFP for VSV). At 24 h after infection, GFP intensity was quantified by flow cytometry as a surrogate marker for viral replication. Zotatifin treatment resulted in a significant reduction in GFP intensity, regardless of the virus or MOI tested (**Figures 6A, B, E, F**), indicating robust inhibition of viral replication. To corroborate the flow cytometry findings, we quantified the virus titers in the cell culture supernatant from PR8-GFP- and rVSV-GFP-infected cells, as well as in cell lysates from VV-infected cells. Zotatifin treatment led to a statistically significant reduction in viral titers for all three viruses (**Figures 6C, D, G–I**).

**Figure 7:**
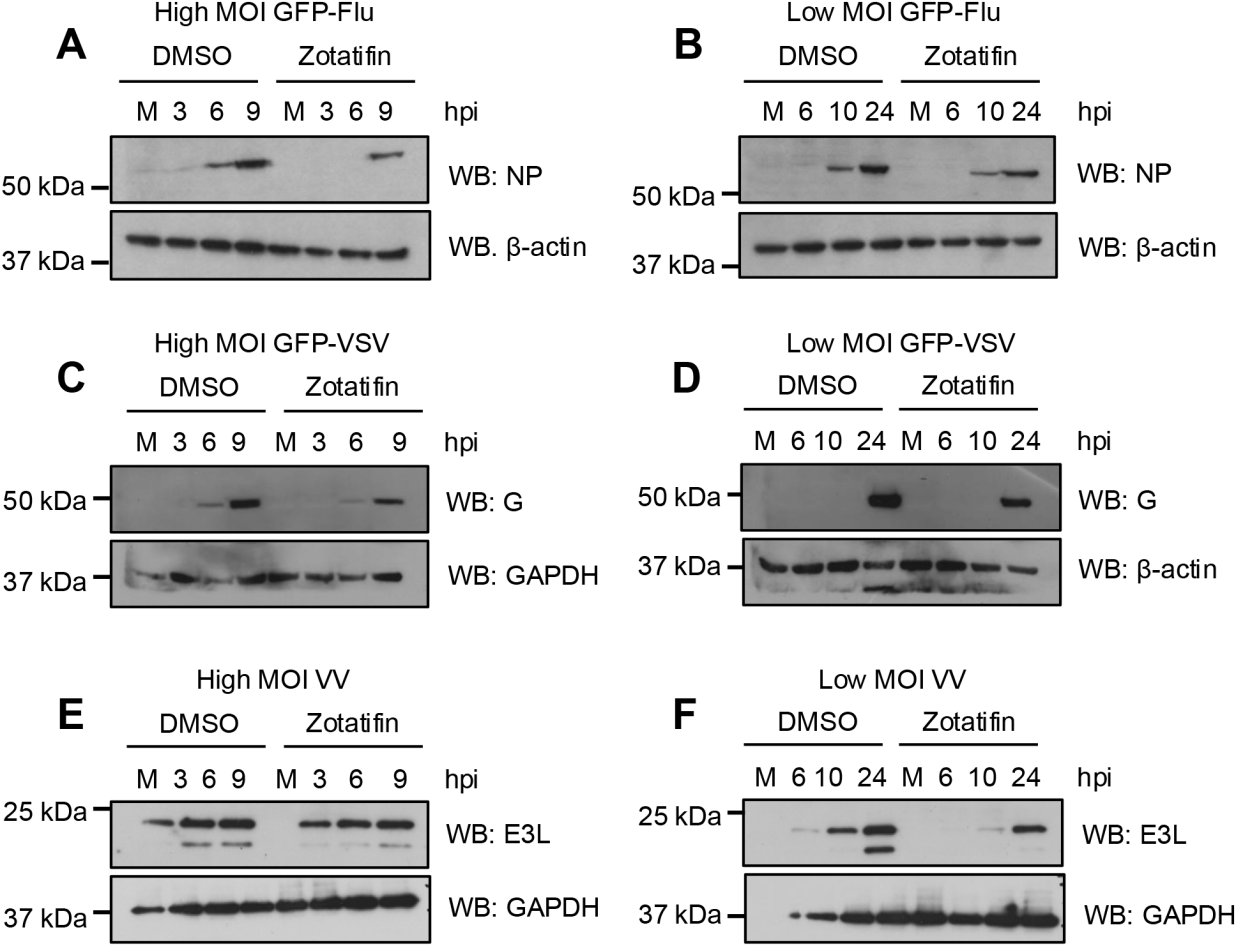
Zotatifin downregulates the synthesis of viral proteins in influenza A virus, vesicular stomatitis virus, and vaccinia virus infected cells. A549 cells were pretreated with zotatifin, then infected with PR8-GFP **(A, B)**, rVSV-GFP **(C, D)**, or vaccinia **(E, F)** viruses, as previously indicated. At the indicated hours after infection, levels of influenza A NP, VSV G, or vaccinia E3L proteins were evaluated using Western blot analysis. β-actin or GAPDH proteins were used as a loading controls. Representative images from three independent experiments are shown.

Finally, we analyzed the effect of zotatifin on viral protein synthesis. We observed a reduction in influenza A, VSV, and vaccinia protein levels in those cells treated with zotatifin, regardless of the MOI tested (**Figures 7A–F**). These findings suggest that zotatifin exhibits an antiviral activity against IAV, VSV, and vaccinia viruses.

## 4 Discussion

The inhibition of eIF4A by the natural rocaglate silvestrol has been reported to exhibit antiviral activity against ZIKV, human rhinovirus, coronavirus, EBOV, and picornavirus (Biedenkopf et al., 2017; Elgner et al., 2018; Muller et al., 2018). Zotatifin, a synthetic rocaglate, exhibits significant antiviral activity against various coronaviruses, including HCoV-229E, MERS-CoV, and SARS-CoV-2 (Ernst et al., 2020; Gordon et al., 2020; Obermann et al., 2022). However, zotatifin’s potential for broad-spectrum antiviral activity remains incompletely characterized.

In this study, we evaluated zotatifin’s potential to control the replication of viruses from five families: *Orthomyxoviridae*, *Rhabdoviridae*, *Flaviviridae*, *Togaviridae*, and *Poxviridae*. Our results revealed that, compared with the rocaglates rocaglamide A and CR-1-31-B, zotatifin exhibits reduced cytotoxicity on HDFs and HMC3 cells, which is consistent with the reduced cytotoxic effects on immune cells as reported previously (Obermann et al., 2022). Zotatifin potently reduces both MAYV replication and viral protein synthesis in a dose-dependent manner. This was demonstrated for three cell lines and different MAYV isolates. Additionally, we demonstrated that zotatifin effectively controls the replication of other togaviruses, including CHIKV and UNAV, and inhibits the medically relevant ZIKV flavivirus. These findings suggest that zotatifin exhibits antiviral activity against diverse arboviruses with similar epidemiological characteristics, thus expanding the therapeutic potential of this compound. Importantly, we demonstrated that zotatifin also downregulates the replication and protein synthesis of other, unrelated viruses, such as influenza A virus, vesicular stomatitis virus, and vaccinia virus, confirming its broad-spectrum antiviral properties as suggested previously (Taroncher-Oldenburg et al., 2021).

Time-of-addition studies revealed that zotatifin administration 2 h post-infection effectively reduced MAYV replication, indicating a robust post-entry antiviral effect. This observation is consistent with the compound’s mechanism of inhibiting protein translation through targeting the host helicase eIF4A (Ernst et al., 2020; Obermann et al., 2022). Notably, zotatifin demonstrated a therapeutic window extending up to 4 h post-infection, which distinguishes it from entry inhibitors and strongly supports its potential as a therapeutic antiviral agent. Additionally, we demonstrated that pretreating cells with zotatifin for just 2 h provides antiviral protection against MAYV. Beyond its direct antiviral effects, our data demonstrate that IFN and IFN-response genes are significantly upregulated in both A549 and HMC3 cells following zotatifin treatment. The robust induction of *ISG15* and *MxA*—two well-characterized antiviral effector genes— (Haller and Kochs, 2011; Morales and Lenschow, 2013) along with the increasing of *DDX58* (*RIG-I*), a pattern recognition receptor that detects viral RNA (Chan and Gack, 2015), suggests that zotatifin may enhance cellular surveillance mechanisms. These findings, combined with previous reports demonstrating that zotatifin induces the IFN pathway (Zhao et al., 2023), led us to propose that zotatifin’s antiviral activity may involve a dual mechanism encompassing both direct translation inhibition and innate immune activation. However, it is likely that additional host cellular pathways also contribute to zotatifin’s antiviral effects (Chen et al., 2021; Ho et al., 2021).

Although zotatifin shows promise as an antiviral treatment, several limitations must be recognized. Our studies were performed exclusively *in vitro*, which may not fully recapitulate the complexity of viral infections *in vivo*. Moreover, the tested viruses represent only a subset of medically important pathogens, limiting the generalizability of our findings. Another important factor to consider is zotatifin’s toxicity profile, which has been characterized in both preclinical and clinical studies.

Zotatifin’s effects have been evaluated in several mouse models due to its potent antitumor activity (Gerson-Gurwitz et al., 2021; Manara et al., 2024). These preclinical studies demonstrated a favorable safety profile, with no evidence of toxicity in animals treated with therapeutically effective doses. No severe adverse effects were reported, and tolerability remained good even when zotatifin was administered with other therapeutic agents (Gerson-Gurwitz et al., 2021; Manara et al., 2024).

In the first human study, zotatifin was administered to patients with advanced solid tumors (Meric-Bernstam et al., 2022). The most common adverse effects included fatigue, anemia, diarrhea, and dyspnea, predominantly of grade 1 or 2 severity. Dose-limiting toxicities comprised grade 2–3 thrombocytopenia and anemia. The maximum tolerated dose was established at 0.07 mg/kg administered over a two-week treatment period followed by one week of rest. Pharmacokinetic analysis revealed a linear, dose-proportional profile, and the adverse effects were generally manageable (Meric-Bernstam et al., 2022).

To our knowledge, this represents the first comprehensive demonstration of zotatifin’s broad-spectrum antiviral activity across multiple virus families with diverse genome types and replication strategies. Zotatifin exhibited consistent antiviral activity against diverse MAYV strains originating from distinct geographic locations, suggesting that this compound may remain effective against genetically variable viruses, a critical consideration in the context of emerging and rapidly evolving viral threats. Furthermore, zotatifin’s broad-spectrum antiviral activity, coupled with its favorable safety profile demonstrated in both preclinical and clinical studies (Boyer et al., 2025; Gerson-Gurwitz et al., 2021; Kuzuoglu-Ozturk et al., 2025; Manara et al., 2025; Meric-Bernstam et al., 2022), supports its potential as a therapeutic candidate for emerging and reemerging viral diseases.

In conclusion, this study comprehensively demonstrates that zotatifin possesses broad-spectrum antiviral activity against multiple viral families (*Togaviridae*, *Flaviviridae*, *Orthomyxoviridae*, *Rhabdoviridae*, and *Poxviridae*), supporting its development as a versatile therapeutic agent for current and future viral threats.

## Data Availability

The original contributions presented in the study are included in the article/**Supplementary Material**. Further inquiries can be directed to the corresponding author.
