# Supplementary material for: Broad-spectrum antiviral activity of the synthetic rocaglate zotatifin against Mayaro virus and other viruses
Source: Front Cell Infect Microbiol. 2026 Mar 19;16:1752166. doi: 10.3389/fcimb.2026.1752166 (PMC13043648; doi:10.3389/fcimb.2026.1752166)
Supplement: Supplementary file 1 [file DataSheet1.pdf]

Supplementary Material

Broad-spectrum antiviral activity of the synthetic rocaglate zotatifin against Mayaro virus and other viruses

Patricia Valdés-Torres[1,2], Dalkiria Campos[1,3], Paola Elaine Galán-Jurado[1], Dalel Zegarra[1,2], Isaac Tuñon-Lorenzo[1], Félix González-Castillo[1], María Blanquer[4], Juan Castillo Mewa[5], Carmen Rivas[4,6*†] and José González-Santamaría[1*†]

[1]Grupo de Biología Celular y Molecular de Arbovirus, Departamento de Investigación en Virología y Biotecnología, Instituto Conmemorativo Gorgas de Estudios de la Salud, 0816-02593, Panamá, Panama

[2]Programa de Maestría en Microbiología Ambiental, Universidad de Panamá, Panamá, Panama

[3]Programa de Desarrollo de las Ciencias Básicas (PEDECIBA), Universidad de la República, Montevideo, Uruguay

[4]Centro de Investigación en Medicina Molecular y Enfermedades Crónicas (CIMUS), Universidad de Santiago de Compostela, 15706, Santiago de Compostela, Spain

[5]Departamento de Investigación en Genómica y Proteómica, Instituto Conmemorativo Gorgas de Estudios de la Salud, Panamá, Panama

[6]Departamento de Biología Molecular y Celular, Centro Nacional de Biotecnología (CNB), CSIC, 29049, Madrid, Spain

*** Correspondence:**
Carmen Rivas and José González-Santamaría
mcarmen.rivas@usc.es; jgonzalezsantamaria@gorgas.gob.pa

†These authors have contributed equally.

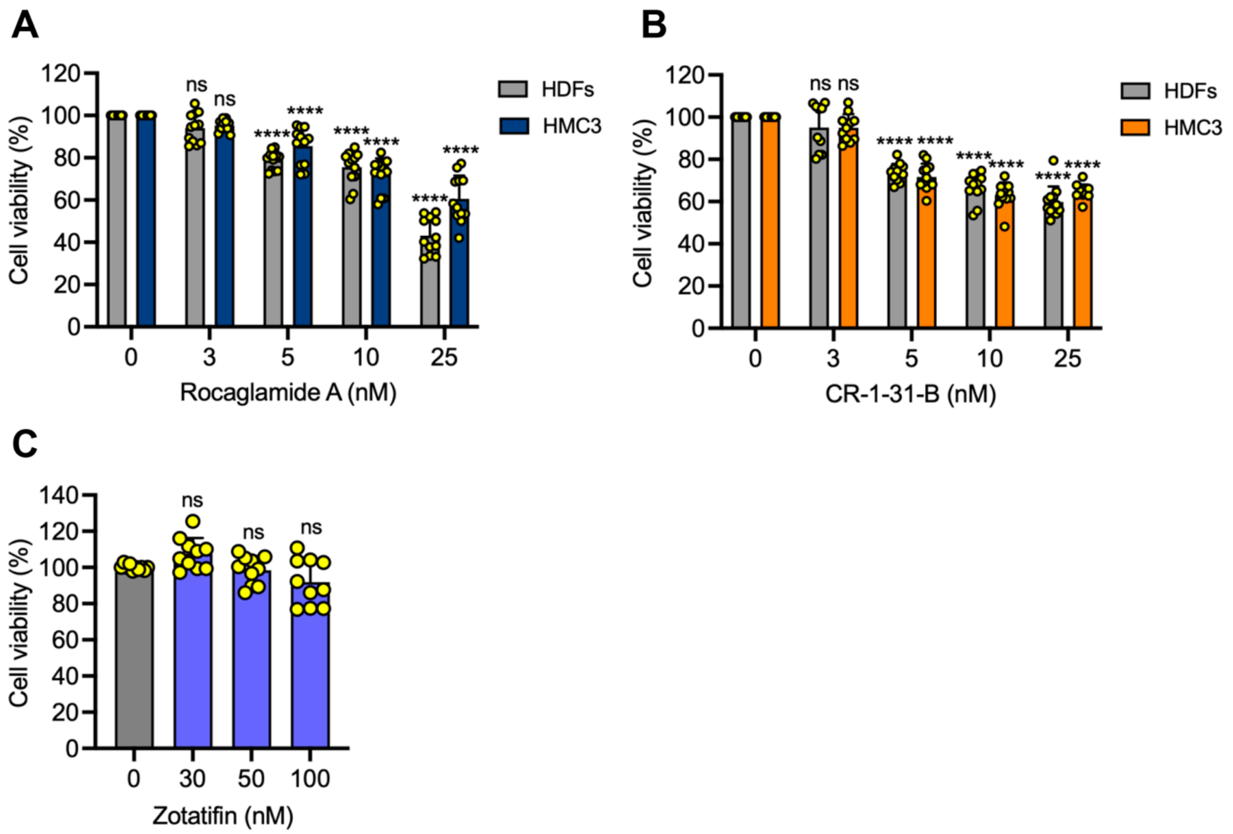

Supplementary Figure 1. Cytotoxicity of the rocaglates in HDFs, HMC3 and A549 cells. (**A-C**) HDFs, HMC3, or A549 cells were treated with the rocaglates at the indicated concentrations. After 24 h of treatment, cell viability was determined using the MTT method. Data represent mean ± standard deviation from two independent experiments, each with five replicates. Statistical analysis was performed using a one-way ANOVA followed by a Dunnett's post hoc test. Significance levels: ns, not significant; ****, $p < 0.0001$.

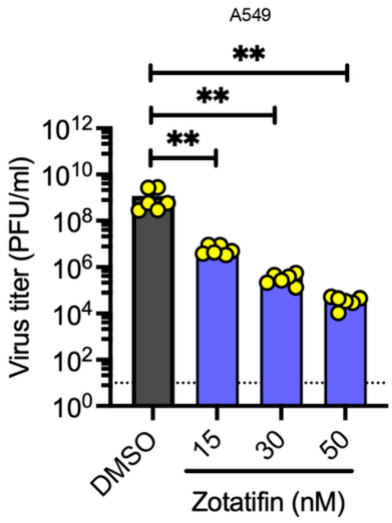

Supplementary Figure 2. Post-infection zotatifin treatment inhibits MAYV replication in A549 cells. A549 cells were infected with MAYV (AVR065 strain, MOI = 1) and incubated 1 h to allow viral adsorption. Following the adsorption period, cells were treated with 50 nM zotatifin and cultured for 24 h post-infection. Viral titers in cells supernatants were determined using plaque-forming assay. The dashed horizontal line indicates the assay limit of detection. Data represent mean ± standard deviation from two independent experiments performed in triplicate. Statistical analysis was performed using a one-way ANOVA followed by a Dunnett´s post hoc test. Significance level: **, $p < 0.01$.

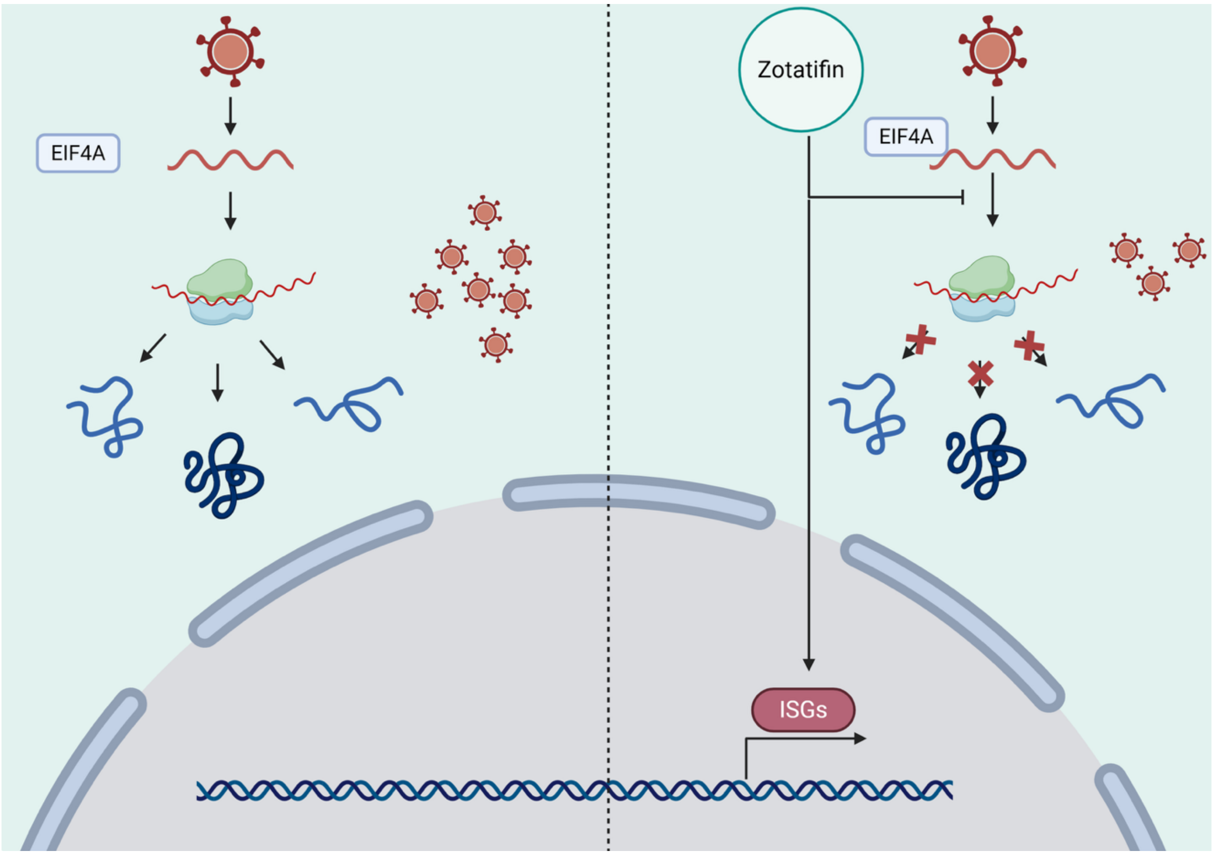

Supplementary Figure 3. Proposed dual mechanisms of zotatifin's antiviral activity. Zotatifin exerts antiviral affects through two complementary mechanisms: (1) **Direct viral inhibition** via targeting eukaryotic initiation factor 4A (eIF4A), thereby blocking viral protein synthesis; and (2) **Indirect immune modulation** through induction of the type I interferon signaling pathway, which enhances the host antiviral response. This graphic was created in BioRender. Rivas, C. (2026) http://BioRender.com/ivrwt44.
